# Editorial: Hydrogenase: structure, function, maturation, and application

**DOI:** 10.3389/fmicb.2023.1284540

**Published:** 2023-09-22

**Authors:** Stefan Frielingsdorf, Constanze Pinske, Francesca Valetti, Chris Greening

**Affiliations:** ^1^Institute of Chemistry, Biophysical Chemistry, Technische Universität Berlin, Berlin, Germany; ^2^Institute for Biology, Microbiology, Martin-Luther University Halle-Wittenberg, Halle (Saale), Germany; ^3^Department of Life Sciences and Systems Biology, University of Torino, Turin, Italy; ^4^Department of Microbiology, Monash University, Clayton, VIC, Australia

**Keywords:** hydrogenase, metalloenzyme, hydrogen, metabolism, biotechnology, metal cofactor maturation

Molecular hydrogen (H_2_) is an important molecule in the metabolism of diverse microorganisms (Greening et al., 2015). Many microorganisms use H_2_ as an electron donor to support aerobic respiration, anaerobic respiration, and carbon fixation. Others produce H_2_ as a means to dispose reducing equivalents through fermentative and photobiological processes. Furthermore, exchange of H_2_ between different species shapes the ecology and biogeochemistry of diverse ecosystems globally (Schwartz et al., 2013). The enzymes that split or evolve H_2_ are called hydrogenases and these metalloproteins can be divided into three phylogenetically unrelated classes distinguishable by the metal composition of their active sites, namely [Fe]-, [FeFe]-, and [NiFe]-hydrogenases (Lubitz et al., 2014). Following a century of hydrogenase research, it is now possible to isolate, handle, and investigate these fragile enzymes. There have been numerous advances in understanding the regulation, function, structures, and maturation of these enzymes, as well as their involvement in important processes such as microbial pathogenesis and greenhouse gas cycling (Lubitz and Tumas, 2007; Greening et al., 2019; Benoit et al., 2020; Ehhalt and Rohrer, 2022). The employment of hydrogenases and hydrogenase-based applications could also potentially facilitate the world's transition to a sustainable H_2_-based energy economy in the future.

Hydrogenases mediate a seemingly simple reaction, i.e., the splitting of H_2_ into a proton and a hydride, followed by the complete separation of protons and electrons. However, the reaction mechanisms of the different enzyme types are not yet fully understood. Likewise, the intricate maturation of the metal cofactors is a constant resource of unprecedented biological chemistry (Bortolus et al., 2018; Britt et al., 2020; Schulz et al., 2020; Arlt et al., 2021; Stripp et al., 2021; Pagnier et al., 2022; Arriaza-Gallardo et al., 2023; Caserta et al., 2023). Additionally, features such as the frequent membrane association and O_2_ sensitivity of hydrogenases has so far hindered *in vitro* harnessing of their function. New structural, spectroscopic, and electrochemical data and methods will enable new insights into their maturation and reactions. In addition, the discovery and characterization of diverse hydrogenases occurring in different microorganisms provide opportunities to discover new physiological roles, structural adaptations, and potential applications of these enzymes (Peters et al., 2015; Dragomirova et al., 2018; Schuchmann et al., 2018; Pinske, 2019; Winkler et al., 2021). Attempts to employ hydrogenases for industrial purposes have been made, however, there is presumably still a long way to go until processes involving hydrogenases (biological H_2_ production or biological fuel cells) will become economically feasible (Rögner, 2015; Ji et al., 2023; Xuan et al., 2023).

This Research Topic comprises 11 articles that aimed to bring together recent advances in hydrogenase research, including structure, function, maturation, and application. In terms of structural investigation, Dragelj et al. theoretically and experimentally analyzed an isolated large subunit of a [NiFe]-hydrogenase. They confirmed that [NiFe] large subunits dimerize in the absence of their small subunit (Hartmann et al., 2018; Kwon et al., 2018; Caserta et al., 2020), and showed that the [NiFe]-cofactor is stabilized by the dimerization. In the realm of structure-function relationships, Kisgeropoulos et al. investigated the effect of amino acid exchanges of the primary proteinaceous proton acceptor in [FeFe]-hydrogenase. The data expand previous work (Cornish et al., 2011; Morra et al., 2012; Duan et al., 2018; Artz et al., 2020), demonstrating that the p*K*_a_ of the proton acceptor is one factor controlling the catalytic bias of the enzyme. Three further articles dealt with functional aspects of hydrogenases. Morra reviewed the identification and characterization of novel [FeFe]-hydrogenases, described their functional roles, and recent findings regarding their O_2_ tolerance. Kpebe et al. reported on the essential role of a bifurcating [FeFe]-hydrogenase in the sulfate reducer *Solidesulfovibrio fructosivorans*, showing that during ethanol oxidation, the hydrogenase confurcates electrons from NADH and reduced ferredoxin to produce H_2_, which is later on used for energy conservation using sulfate as electron acceptor. The function of a bidirectional [NiFe]-hydrogenase in *Synechocystis* sp. PCC 6803 was investigated by Burgstaller et al.. They showed that this hydrogenase is essential for growth on arginine and glucose in the presence of light and O_2_, and interestingly this function seems to be unrelated to H_2_ catalysis. Instead, an H_2_-independent role for the transfer of electrons into the photosynthetic electron transfer chain is proposed, which is an important hypothesis that may also be relevant for certain multisubunit hydrogenases in other species.

On the subtopic of hydrogenase maturation, Haase and Sawers investigated residues that contribute to [NiFe]-hydrogenase maturation. They found a histidine residue in a HypC-type chaperone that is important for efficient binding to its maturation partner HypD (Blokesch and Böck, 2002). In addition, this residue is important for selectivity, ensuring that only the correct large [NiFe]-hydrogenase subunit is matured. It should be emphasized that understanding cofactor maturation and incorporation is of high importance for any future application of hydrogenases, which is demonstrated in the following article. In an interdisciplinary approach in the fields of maturation and application, Fan et al. significantly improved the [NiFe]-cofactor incorporation in the course of heterologous production of a [NiFe]-hydrogenase from *Cupriavidus necator* in *Escherichia coli*. Previously, cofactor insertion was not functional, resulting in production of inactive hydrogenases (Fan et al., 2021).

Another four articles explore the application of hydrogenases. Hogendoorn et al. isolated the novel strain “*Candidatus* Hydrogenisulfobacillus filiaventi” R50 gen. nov., sp., nov. which was characterized as a chemolithoautotrophic thermoacidophilic aerobic H_2_-oxidizing bacterium. This strain excretes about half of the fixed CO_2_ in the form of amino acids, which makes it a promising candidate for the industrial production of organic compounds from CO_2_, using H_2_ as energy source. H_2_ oxidation in this strain is facilitated by two [NiFe]-hydrogenases from the groups 1b and 1h, with the latter conferring high affinity toward H_2_ (Søndergaard et al., 2016). Kobayashi et al. engineered the acetogenic bacterium *Moorella thermoacetica* for enhanced acetate, ethanol and acetone production. They found that H_2_ supplementation under mixotrophic conditions increases NADH levels but can inhibit growth due to an unbalanced redox status. This study emphasized the role of a reversible electron-bifurcating group A3 [FeFe]-hydrogenase for balancing the cellular redox state. These observations may also be exploited for biohydrogen production by that strain. Another way to produce H_2_ was analyzed by Barahona et al.. In order to enhance nitrogenase-driven H_2_ production in *Rhodobacter capsulatus*, they used a sensory hydrogenase coupled to the production of a fluorescence signal. By inducing genome-wide mutations and using high-throughput fluorescence-activated cell sorting, they were able to generate mutants with elevated H_2_ evolution capabilities. Finally, Schumann et al. reviewed current state-of-the-art and future perspectives for efficient light-driven H_2_ production using phototrophic microorganisms. This technology potentially enables conversion of solar energy into a chemical compound that enables storage and transport. However, as discussed by the authors, extensive engineering is required for this technology to be efficient and scalable.

Taken together, the Research Topic advances our knowledge especially on the function and application of hydrogenases and provides important perspectives for future H_2_-based biologically green technologies.

## Author contributions

SF: Writing—original draft, Writing—review and editing. CP: Writing—review and editing. FV: Writing—review and editing. CG: Writing—review and editing.
